# Nano-adjuvanted dry powder vaccine for the mucosal immunization against airways pathogens

**DOI:** 10.3389/fvets.2023.1116722

**Published:** 2023-03-14

**Authors:** Elena Canelli, Luca Ferrari, Paolo Borghetti, Francesco Candela, Nkemjika Sopuru Abiakam, Annalisa Bianchera, Francesca Buttini, Gian Enrico Magi, Fabio Sonvico, Paolo Martelli, Ruggero Bettini

**Affiliations:** ^1^Department of Veterinary Science, University of Parma, Parma, Italy; ^2^Advanced Drug Delivery Research Laboratory, Department of Food and Drug, University of Parma, Parma, Italy; ^3^Interdepartmental Centre Biopharmanet-Tec, University of Parma, Parma, Italy; ^4^School of Biosciences and Veterinary Medicine, University of Camerino, Camerino, Italy

**Keywords:** mucosal vaccination, nanoemulsion, chitosan, *Mycoplasma hyopneumoniae*, dry powder, nasal drug delivery

## Abstract

Nasal vaccination has been shown to provide optimal protection against respiratory pathogens. However, mucosal vaccination requires the implementation of specific immunization strategies to improve its effectiveness. Nanotechnology appears a key approach to improve the effectiveness of mucosal vaccines, since several nanomaterials provide mucoadhesion, enhance mucosal permeability, control antigen release and possess adjuvant properties. *Mycoplasma hyopneumoniae* is the main causative agent of enzootic pneumonia in pigs, a respiratory disease responsible for considerable economic losses in the pig farming worldwide. The present work developed, characterized, and tested *in vivo* an innovative dry powder nasal vaccine, obtained from the deposition on a solid carrier of an inactivated antigen and a chitosan-coated nanoemulsion, as an adjuvant. The nanoemulsion was obtained through a low-energy emulsification technique, a method that allowed to achieve nano droplets in the order of 200 nm. The oil phase selected was alpha-tocopherol, sunflower oil, and poly(ethylene glycol) hydroxystearate used as non-ionic tensioactive. The aqueous phase contained chitosan, which provides a positive charge to the emulsion, conferring mucoadhesive properties and favoring interactions with inactivated *M. hyopneumoniae*. Finally, the nanoemulsion was layered with a mild and scalable process onto a suitable solid carrier (*i.e*., lactose, mannitol, or calcium carbonate) to be transformed into a solid dosage form for administration as dry powder. In the experimental study, the nasal vaccine formulation with calcium carbonate was administered to piglets and compared to intramuscular administration of a commercial vaccine and of the dry powder without antigen, aimed at evaluating the ability of IN vaccination to elicit an *in vivo* local immune response and a systemic immune response. Intranasal vaccination was characterized by a significantly higher immune response in the nasal mucosa at 7 days post-vaccination, elicited comparable levels of *Mycoplasma*-specific IFN-γ secreting cells and comparable, if not higher, responsiveness of B cells expressing IgA and IgG in peripheral blood mononuclear cells, with those detected upon a conventional intramuscular immunization. In conclusion, this study illustrates a simple and effective strategy for the development of a dry powder vaccine formulation for nasal administration which could be used as alternative to current parenteral commercial vaccines.

## 1. Introduction

*Mycoplasma hyopneumoniae* (*M. hyopneumoniae*) is the primary pathogen of enzootic pneumonia (EP) in pigs, a respiratory disease occurring worldwide and causing major economic losses to pig farming. Losses are mainly due to increased costs for medication and reduced growth of pigs. The pathogen is also one of the primary agents involved in the porcine respiratory disease complex (PRDC). *M. hyopneumoniae* adheres to and damage the ciliated epithelial cells of the respiratory tract, and the mucosal immune response plays a major role in the prevention and control of the infection (1).

The control of the disease may be achieved through the combination of different strategies, first and foremost proper management practices and housing conditions, together with strategic medication and finally with vaccination (1). Commercially available vaccines against *M. hyopneumoniae*, consisting of inactivated, adjuvanted whole-cell preparations, *i.e*., bacterins, are usually administered by intramuscular injection. However, in general, these vaccines provide only partial clinical protection and do not prevent the colonization of the respiratory tract. Different factors, such as inappropriate vaccine storage and administration techniques, antigenic differences between field strains and vaccine strains, and presence of the disease already at the time of vaccination may negatively influence vaccination efficacy (1, 2).

Being the most likely portal of entry of pathogens into the body, mucosal surfaces are sites of intense immunological activity. In particular, at least theoretically, intranasal vaccination could provide optimal protection against respiratory pathogens, by eliciting both humoral and cell-mediated immunity both locally and systemically (3). Indeed, the nasal-associated lymphoid tissue (NALT) has a full range of immunocompetent cells, including B lymphocytes, CD4+ and CD8+ lymphocytes, phagocytic antigen-presenting cells and various subsets of dendritic cells (4). Consequently, intranasal vaccination could represent a very promising route of administration for mass vaccination in piglets as it efficiently stimulates the mucosal immune response at the respiratory tract level and assures vaccine compliance. Nevertheless, the efficiency of the intranasal administration against *M. hyopneumoniae* needs the development of an appropriate adjuvant and pharmaceutical technology for the delivery and uptake of the antigen.

Several adjuvants and delivery vehicles developed for vaccines are submicron in size; viral vectors, virosomes, immunostimulating complexes (ISCOM) and liposomes are only a few among the nanoscale vectors used for vaccination (5, 6). In the group of these nano-adjuvants, nanoemulsions (NEs), two-phase colloidal dispersions forming either oil-in-water (O/W) or water-in-oil (W/O) systems, stand out as a simple, effective way to increase the immune response, reduce the amount of antigen required and avoid multiple dosing (7–9). In addition, chitosan, a cationic polysaccharide of natural origin, has been recently indicated as one of the most promising excipients for veterinary applications (10–12). Chitosan presents a series of appealing properties as nasal and vaccine excipient, providing mucoadhesion, enhanced mucosal permeability, control of the antigen release and adjuvant effects (13, 14).

Regarding parenteral vaccines, as well as in many of the vaccines under development for mucosal delivery, the formulation is administered in liquid form. However, the use of dry powder formulations has been suggested since they can provide physical, chemical, and microbiological stability to the formulation, potentially avoiding the need for preservatives, buffers, and cold-chain distribution (15).

In this context, the present study aims to develop a new intranasal vaccine for piglets based on *M. hyopneumoniae* bacterins, using as an adjuvant a novel chitosan-modified nanoemulsion, to be administered in form of dry powder. Three different pharmaceutical excipients (lactose, mannitol, and calcium carbonate) in powder form were tested as the inert carrier. The carrier powders were wetted with the nanoemulsion containing the antigen and subsequently dried under controlled conditions.

## 2. Materials and methods

### 2.1. *M. hyopneumoniae* nasal vaccine components

Chitosan, ChitoClear FG95^®^LV from PRIMEX (Siglufjourdur, Iceland) was used as received. The supplier reports a viscosity of 8 cP and 99% degree of deacetylation for the batch used (TM1703). Poly(ethylene glycol) hydroxystearate (PEG 660 12-hydroxystearate, Crodasol™ HS HP, Croda Europe Ltd, Snaith, England), vitamin E (DL-α-tocopherol, A.C.E.F. S.p.A., Fiorenzuola d'Arda, Italy) and sunflower oil (A.C.E.F. S.p.A., Fiorenzuola d'Arda, Italy) were selected to produce NEs.

Three different carrier powders were used for the preparation of the nasal vaccine: lactose (Pharmatose^®^ 325M, DFE Pharma, Goch, Germany), calcium carbonate (Destab^TM^ 90S, Seppic, Puteaux, France) and mannitol (PEARLITOL^®^ 200 DC, Roquette Pharma, Lestrem, France).

A commercial intramuscular vaccine against *M. hyopneumoniae* and *M. hyopneumoniae* bacterins were used for the experimental *in vivo* study. The antigen was provided in culture medium and washed by three successive centrifugations followed by redispersion with ultrapure water. It was then suspended in ultrapure water with a bacterin concentration of 1·10^10^ bacterins/ml and stored at 4°C till needed.

Ultrapure type II water was obtained using a filtration system (resistivity: 0.055 MΩ·cm, PURELAB Pulse, Veolia Water Technologies Italia, Zoppola, Italy) and all other solvents and reagents were at least of analytical grade.

### 2.2. Nanoemulsion preparation

O/W NEs were obtained by a low-energy nano-emulsification process using an oil phase composed of vitamin E, sunflower oil and the non-ionic surfactant Crodasol™ (PEG 660 12-hydroxystearate) and an aqueous phase enriched with chitosan at a concentration of 0.5% w/v. The ratio between the aqueous phase and the oily phase was kept constant at 80:20 throughout the whole process.

The 0.5% w/v chitosan solution (pH = 4) was prepared by adding 0.5 g of chitosan in 100 ml 0.5% w/v acetic acid aqueous solution. This solution was continuously stirred for 6–8 h to dissolve the polysaccharide completely, then filtered (RC Membrane Filters, 0.45 μm, Sartorius, Göttingen, Germany) and stored at room temperature until needed. The main reason for the use of the chitosan solution in the emulsion is to obtain surface modified nanoemulsions in which chitosan could provide positive charge and mucoadhesion properties to the adjuvant nanoemulsion. The aqueous phase represented 80% of the weight of the final preparation. The second phase (the oily phase) was composed of vitamin E, sunflower oil plus the totally miscible non-ionic surfactant Crodasol in a proportion of 1:1:2. The oily phase was kept at 70°C with continuous magnetic stirring for about 1 h to obtain a homogeneous solution.

Finally, to obtain the NEs, the oily phase was slowly poured into the aqueous phase (at 25°C) which was mechanically stirred at 14,420 rpm using a mechanical dispersing device (T10 Standard ULTRA TURRAX^®^ equipped with an S10N−8G dispersing tool, IKA-Werke GmbH & Co. KG, Staufen, Germany).

While the aqueous-to-oily phase ratio was kept constant, within the oily phase, to optimize the average size and particle size distribution of the obtained NE, the surfactant was used at different concentrations, whereas the oils, *i.e*., vitamin E and sunflower oil, were kept in a proportion 1:1. Table 1 reports the surfactant-to-oil weight ratios (SOR, %) used to prepare the nanoemulsions.

**Table 1:** Different proportions (SOR, %) of surfactants and oils used to prepare the nanoemulsions with the low-energy nanoemulsification method.

**Nanoemulsion**	**SOR (%)**	**Surfactant (g)**	Oils^*^(g)
NE30	30	0.6	1.4
NE40	40	0.8	1.3
NE50	50	1	1
NE60	60	1.2	0.8
NE70	70	1.4	0.6

^*^Oils = vitamin E + sunflower oil (1:1).

### 2.3. Nanoemulsion characterization

Particle size measurements of the NE were performed using dynamic light scattering (DLS, Zeta Plus Analyzer, Brookhaven Instruments Ltd, Redditch, UK). The 35 mW red diode laser operates at a nominal 640 nm wavelength with the scatter angle fixed at 90° and the temperature maintained at 25°C. Before particle size measurements, the samples were diluted with ultrapure water to obtain a scattered light intensity of around 100 kcts. The analysis data of the particle sizing and polydispersity were calculated as a mean of three separated batch analyses with its relative standard deviation.

Furthermore, particle size, concentration and aggregation measurements of a selected NE were performed using Nanoparticle Tracking Analysis (NTA). Experiments were conducted using a NanoSight NS300 instrument (Malvern Instruments Ltd., Malvern, UK) equipped with a 480 nm laser light source and a 20 × magnification microscope was used to carry out the particle tracking analysis with a field of view of ~100 × 80 × 10 μm. The built-in sCMOS camera was used to record videos and the particle tracking was analyzed by NTA 3.1 instrument software. The software tracks single particles in Brownian motion through the light they scatter and converts their motion into particle hydrodynamic diameter based on a variation of the Stoke-Einstein equation. Furthermore, knowing the volume of the suspension and the dilution, the associated NTA software is capable to calculate an approximate concentration of the nanoparticles inside the colloidal suspension. The nanoemulsion was highly diluted (1:630,000) with ultrapure water for allowing single particle tracking. After that, the sample was drawn into 1 ml plastic syringe, which was used for full sample injection into the instrument sample chamber. The nanoparticle images were acquired as 60 s videos of the sample three times, which were used for subsequent analysis. Measurement was carried out at a defined temperature (28–28.2°C) and viscosity (0.828–0.832 cP). The result values were obtained as the mean and standard deviation of three runs.

### 2.4. Preparation of the powders loaded with nanoemulsion and antigen

To improve nasal delivery, dry powders loaded with both NE and *M. hyopneumoniae* bacterins were prepared. Three different solid carriers, *i.e*., lactose (Pharmatose^®^ 325M, DFE Pharma, Goch, Germany), calcium carbonate (Destab^TM^ 90S, Seppic, Puteaux, France) and mannitol (PEARLITOL^®^ 200 DC, Roquette Pharma, Lestrem, France) were selected. For each excipient, a particle size fraction between 38 and 106 μm was obtained by sieving (Endecott Sieves, London, UK) to obtain particles with dimensions suitable for nasal administration. The nanoemulsion NE50, formulated at room temperature using 50% w/w of Crodasol™, 25% w/w of vitamin E and 25% w/w of sunflower oil, was solidified on suitable powder carriers thanks to an innovative controlled wetting/drying approach (16). Briefly, the controlled wetting/drying process of the three powders was achieved as follows: the powder was put inside a mortar heated at 45°C and wetted progressively with small quantities (100 μl) of the nanoemulsion using a metering spray pump (Aptar, Le Vaudreuil, France). The powder was then manually mixed with a pestle until most of the liquid was distributed in the powder bed. At each 0.5 g of emulsion added, the powder was left to dry at 45°C for 30 min in a vacuum oven (Gallenkamp Sanyo/Weiss Vacuum Oven, Leicestershire, UK) to dry the excess water present. The operation was repeated until the required loading was obtained.

Initially, three batches were prepared for each excipient (mannitol, calcium carbonate, and lactose), the carrier powder amount was maintained constant at 2 g, while the quantity of liquid nanoemulsion added was 2, 3 or 4 g to obtain 9 powder formulations coded as reported in Table 2.

**Table 2:** Nanoemulsions/carrier powders obtained using a controlled wetting/drying process.

**Formulation**	**Carrier type**	**Carrier amount (g)**	**Nanoemulsion amount (g)**	**Nanoemulsion/carrier** **weight ratio**
MN1	Mannitol	2	2	1
MN1.5	Mannitol	2	3	1.5
MN2	Mannitol	2	4	2
CN1	Ca Carbonate	2	2	1
CN1.5	Ca Carbonate	2	3	1.5
CN2	Ca Carbonate	2	4	2
LN1	Lactose	2	2	1
LN1.5	Lactose	2	3	1.5
LN2	Lactose	2	4	2

The morphology of the NE-loaded powders obtained was studied using a Field Emission Scanning Electron Microscope (FESEM Auriga Compact, Zeiss, Jena, Germany). The samples were prepared by dispersing a few milligrams of powder directly onto the carbon tape placed on aluminum stubs. The surface morphology and texture of the particles were investigated in plan-view by using a 1 kV electron beam acceleration voltage. Such a low value allowed to analyze the microparticles without the need for metallization. All the images were acquired with the Everhart-Thornley detector for secondary electrons.

NE-loaded powders were then analyzed for the capacity to restore the nanoemulsion after dispersion in aqueous media. Briefly, 0.5 g of each powder were dispersed in 10 ml of ultrapure water and left overnight without stirring. After redispersion for particle size assessment, the samples were centrifuged for 5 min at 2,000 × g (Microcentrifuge, Scilogex, Rocky Hill, CT, USA) to separate possible undissolved powder. The supernatant was then collected and further diluted with ultrapure water in a proportion of 1:3 for particle size analysis using DLS as described in Section 2.3.

Powders loaded with both nanoemulsion and *M. hyopneumoniae* bacterins were prepared using a selected formulation based on the above-described experiments. A mixture of inactivated whole-cell concentrate of *M. hyopneumoniae* and the nanoemulsion NE50 was obtained under stirring at room temperature. The liquid mixture was then used to coat mannitol (NVP1) or calcium carbonate (NVP2), selected as solid carriers. The liquid dispersion of submicron particulate adjuvant and antigen was deposited onto the solid carrier employing the controlled wetting/drying process described above, using a liquid-to-solid ratio of 2:1.

To assess whether it was still possible to redisperse both nanoemulsion and antigen, 0.4 g of powders were dispersed in 2 ml of ultrapure water and further diluted with a further 70 ml before analysis by laser diffraction performed using the wet dispersion system of the Spraytec instrument (Malvern Instruments Ltd, UK).

All the experiments were replicated at least three times and results expressed as mean value and standard deviation.

### 2.5. Characterization of the performance of a nasal powder device

In view of the administration *in vivo*, the NE-loaded powders were then characterized for their ability to be emitted with a nasal powder device (Monodose Nasal Insufflator, MIAT, Milano, Italy; see Supplementary material, Supplementary Figure 1). This device is a reusable device that works with capsules (HPMC, size 3, Qualicaps, Alcobendas, Spain) loaded with a pre-dosed amount of powder. The capsule must be positioned inside the insufflator and pierced at both ends with needles. Then the capsule is oriented toward the nasal adapter tip and the powder is extracted from the capsule and aerosolized by the airflow generated by manually squeezing the device rubber bulb (puffing).

Capsules were loaded with accurately weighed 25 mg of each powder tested. The amount of powder delivered after each puff (total emitted dose, TED) was measured by weighing the insufflator before and after the squeeze (Crystal 500 SMI, Gibertini, Novate Milanese, Italy). The measurements of the emitted dose were taken after each puff for a total of three puffs. The test was repeated three times for each powder and the results are reported as mean of the sampling with their relative standard deviation. Three capsules for each type of powder were tested on the same day of their preparation. Other capsules were prepared, packaged in heat-sealed aluminum pouches, and stored under controlled conditions at 25°C as well as at 45°C with 75% relative humidity. Capsules stored at 25°C were tested after 30 days measuring the amount of powder emitted (TED) with the MIAT nasal device, while those stored at 45°C were tested after 14 days.

### 2.6. Experimental *in vivo* study

An experimental study was carried out to demonstrate the ability of the intranasally (IN) administered dry powder *M. hyopneumoniae* vaccine to induce an *in vivo* local immune response, and a specific cellular immune response in piglets in comparison with a conventional formulation administered intramuscularly (IM).

Eighteen 28-day-old healthy weaned piglets derived from a conventional farm, free from porcine reproductive and respiratory syndrome virus (PRRSV), porcine circovirus type 2 (PCV2), *M. hyopneumoniae*, and swine influenza virus (SIV), were randomly ear-tagged and assigned to 3 groups in separate pens: IN group (*N* = 8) intranasally administered with the experimental *M. hyopneumoniae* vaccine formulation, IM group (*N* = 6) intramuscularly administered with a commercially available inactivated *M. hyopneumoniae* bacterin-based vaccine (considered as positive control group) and C group administered with the dry powder of the experimental vaccine without the antigen (negative control/mock-vaccinated group) (*N* = 4). The animals were tested for the above-mentioned pathogens before the enrollment and resulted negative (PCR from nasal swabs was performed to check for *M. hyopneumoniae* and SIV, and from blood for PRRSV and PCV2).

The study was carried out at the Department of Veterinary Science, University of Parma, Italy, according to the authorizations provided by the Ethical Committee (PROT. N. 46/OPBA/2017) and by the Italian Ministry of Health (Sper. Min. Aut. 519/2017-PR).

The nasal administration was carried out by using the dry powder vaccine obtained by drying the submicron particulate adjuvant mixed with the *M. hyopneumoniae* antigen (kindly provided by the same manufacturer of the commercial IM vaccine) onto a solid carrier according to the procedure presented in Section 2.4. For this specific experiment, the submicron particulate adjuvant used was NE50, an O/W nanoemulsion prepared using PEG 660 12-hydroxystearate as surfactant and an SOR of 1:1 (50%) as described in Table 1.

The mixture between the inactivated whole-cell concentrate of *M. hyopneumoniae* (1·10^10^ bacterins/ml in water, stored at 4°C) and the nanoemulsion in a volume ratio of 66:33 (NE-*M. hyopneumoniae*) was obtained under stirring at room temperature. The solid carrier selected was calcium carbonate (Destab^TM^ 90S, Seppic, Puteaux, France).

To produce the nasal vaccine powder (NVP2), the liquid dispersion of submicron particulate adjuvant and antigen was deposited onto the solid carrier using the controlled wetting/drying process described above (Section 2.4), with a liquid-to-solid ratio of 2.

Dry powder vaccine NVP2 was administered to piglets of the IN group by using the Monodose Nasal Insufflator (MIAT, Milano, Italy). Two capsules containing each 25 mg dry powder vaccine NVP2 were administered to each piglet, one for each nostril (see Supplementary Figures 1, 2).

Nasal mucosa tissue samples were collected from piglets humanely euthanized on days 2, 7, and 47 post-vaccination (PV). Specifically, two IN, one IM, and two C piglets were euthanized at 2 days PV; two IN, one IM, and two C piglets were euthanized at 7 days PV; all remaining IN (four) and IM (four) piglets were euthanized at 47 days PV.

The method used for euthanasia was an overdose of anesthetic. This method is included in the Annex IV of the European Directive 2010/63/EU. A sodium thiopental (barbiturate) overdose was used for euthanasia (200 mg/kg b.w.), by IV (intravenous) inoculation, after appropriate sedation with azaperone (intramuscular administration at a dose of 1 mg/kg b.w.).

Peripheral blood samples in Li-heparin were collected at 0, 14, 21, 28, 35, and 47 days PV to evaluate the systemic immune responsiveness of T cells and B cells by means of an IFN-γ ELISPOT assay and flow cytometry, respectively. In line with the results reported by Soldevila et al. (17) regarding the quantification of immune cell frequencies, the mean value of the mock-vaccinated control group at 0 days PV was used as the reference value for group comparisons throughout the study period.

#### 2.6.1. Histological analysis

For sampling the nasal mucosa, 4 biopsies were obtained for each animal, two for each nasal cavity (left and right). The samples were fixed in 10% neutral buffered formalin for 48 h, then washed in running water, subsequently dehydrated in an increasing alcohol scale and clarified by using an automatic tissue processor. The biopsies were embedded in paraffin blocks. 3 μm-thick sections were obtained from each biopsy and stained with hematoxylin and eosin (H&E) using an automatic stainer. The sections were examined by optical microscopy (Leica DMRB, Leica Microsystems, Wetzlar, Germany) and images were acquired by a Leica camera.

Every single section, representative of a biopsy, was entirely analyzed at low (4–10 × ) and medium (20 × ) magnification and was attributed an overall histological score using semi-quantitative criteria, according to Salvaggio et al. with modifications (18) including the following histological parameters: (a) the percentage of mucosal involvement by the histological changes considering the entire biopsy (0–4); (b) the localization of the immune/inflammatory cells (0–3); (c) the degree of severity of the immune/inflammatory cell infiltrates (0–3); (d) the glandular damage (0–3); and (e) the epithelial damage (Supplementary Table 1). The immune/inflammatory cells in the infiltrate (neutrophils, eosinophils, lymphocytes, plasma cells, macrophages, and mast cells) were also evaluated by using a semi-quantitative score: – (absent), + (few < 5%), ++ (between 5 and 25%), +++ (between 25 and 50%), ++++ (more than 50%).

#### 2.6.2. IFN-γ ELISPOT for *M. hyopneumoniae*

The T cell immune response was quantified in terms of frequencies of *M. hyopneumoniae*-specific interferon-gamma secreting cells (IFN-γ SC) in porcine peripheral blood mononuclear cells (PBMC) by ELISPOT as previously described (19). Briefly, PBMC were isolated by Histopaque-1077^®^ density gradient (Sigma-Aldrich, St. Louis, MO, USA) and plated at a density of 8·10^5^ cells/well in complete RPMI-1640 (cRPMI-1640) medium + 10% fetal bovine serum (FBS) in 96-well-plates (MultiScreen HTS-IP, Millipore, Billerica, MA, USA) previously coated overnight at 4°C with 10 μg/ml anti-pig IFN-γ mAb (clone P2G10, BD Pharmingen, Franklin Lakes, NJ, USA), washed 4 times with sterile PBS, and then incubated for 2 h at 37°C with cRPMI-1640 + 10% FBS (blocking step). After discarding the blocking solution, the plated cells were stimulated with the inactivated *M. hyopneumoniae* antigen at 100 bacterins/ml (~50 μg/ml) (20) in cRPMI-1640 + 10% FBS for 20 h. After discarding the cells, the spots due to IFN-γ secretion by stimulated individual cells were detected by sequential incubation with 0.5 μg/ml anti-pig IFN-γ biotin-labeled mAb (clone P2C11, BD Pharmingen, Franklin Lakes, NJ, USA) and 1:750 AP-conjugated anti-biotin mAb (Vector Labs, Burlingame, CA, USA) in PBS + 0.5% bovine serum albumin (BSA). Plates were finally incubated with BCIP/NBT (BioRad, Hercules, CA, USA) and the colorimetric reaction was stopped with distilled water. The number of spots in each well-corresponding to the number of *M. hyopneumoniae*-specific IFN-γ SC was quantified using an AID^®^ ELISpot Reader and AID^®^ ELISPOT software v.6.0 (Autoimmun Diagnostika, Strassberg, Germany). As positive control, 4·10^5^ PBMC/well were incubated with phytohemagglutinin (PHA, 10 μg/ml) while, as negative control, 8·10^5^ PBMC were incubated without antigen. The mean number of spots in two negative control wells for each sample was subtracted from the respective mean count in two wells containing stimulated cells and the resulting immune response was expressed as number of IFN-γ SC/10^6^ PBMC (IFN-γ SC frequency).

#### 2.6.3. Quantification of IgA+ and IgG+ B cells upon *M. hyopneumoniae* stimulation

B cell immune responsiveness was determined by quantifying the percentages of B cells expressing surface IgA or IgG in PBMC upon *in vitro M. hyopneumoniae* antigen stimulation using flow cytometry as previously described (21). Briefly, PBMC were plated at 3·10^6^ cells/ml in 24-well-plates in cRPMI-1640 + 10% FBS and stimulated for 44 h at 37°C, 5% CO_2_, with the *M. hyopneumoniae* antigen used for ELISPOT or kept unstimulated as negative control. The negative control values were subtracted from the respective counts of the stimulated cells and the immune response was expressed as percentage of CD79α+IgA+ and CD79α+IgG+ cells.

Cell number and viability were determined after the incubation period by optical microscopy and Trypan blue. Cells were first stained with 1/4,000 LIVE/DEAD^®^ Fixable Far Red Dead Cell Stain kit (Invitrogen Thermo Fisher Scientific, Paisley, UK) in the dark, for 30 min, to exclude dead cells from flow cytometry analysis, washed and stained with primary mouse anti-pig IgA (clone K611B4, IgG_1_, BioRad) or mouse anti-pig IgG (clone F007-1241, IgG_1_, BD Pharmingen, Franklin Lakes, NJ, USA) for 15 min. Secondary goat anti-mouse IgG_1_-FITC (1070-02, Southern Biotech, Birmingham, AL, USA) was used for both primary antibodies, incubating cells for 15 min at 4°C, in the dark. Cells were fixed (15 min, room temperature, in the dark) and permeabilized (50 min, room temperature, in the dark) using Leucoperm™ kit solutions (BioRad). Intracellular staining of CD79α was performed using a mouse anti-human CD79α-PE cross-reactive antibody (clone HM57, IgG_1_, BioRad) (22). Unstained PBMC or PBMC incubated with secondary Ab were used as negative controls. FMO controls were also performed for each staining combination. The analysis was performed using a Cytomics FC500 flow cytometer and CXP software (Beckman-Coulter, Indianapolis, IN, USA) based on singlets, live cells, and lymphocyte gating after the acquisition of at least 40,000 events.

### 2.7. Statistical analysis

Statistical analyses were carried out by using Analysis of Variance (ANOVA) with group, sampling time, and interaction between group and sampling time as fixed factors. Differences among groups at each time point and over time within the group were considered significant when *p* < 0.05. Statistical analyses were carried out using SPSS v.26.0 (SPSS Statistics, IBM, Armonk, NY, USA). Data regarding ELISPOT and flow cytometry analyses are presented as means ± standard deviation.

With specific regard to the experimental design, pigs were assigned to each group by simple randomization.

Assuming a normalized mean of the 2 control groups to 1 and considering a SD per group of about 0.4, reasonable value in the absence of outliers, *N* = 4 animals per group were sufficient to detect in the IN-treated group a doubling in the mean value compared to the other 2 groups [alpha = 0.05; power = 1-beta = 0.8]. For all variables followed over time, point differences at all time points were evaluated by one-way ANOVA, followed by *post-hoc* Tukey's tests.

Histological data were analyzed using GraphPad Prism 9 software (GraphPad Software Inc., La Jolla, CA, USA). Data are expressed as median values by using box and whisker plots. For the overall histological score and for each single histological parameter at 2 and 7 days post-vaccination, a Kruskal-Wallis test followed by a Dunn's multiple comparisons test was used to analyze differences among the three groups. At 47 days post-vaccination, differences between the IN group and the IM group for the overall histological score and for each histological parameter were evaluated by using a Mann-Whitney test in accordance with previously published histological scoring data (23). A *p* < 0.05 was considered significant.

## 3. Results

### 3.1. Development of an O/W nanoemulsion adjuvant

**Figure 1:**
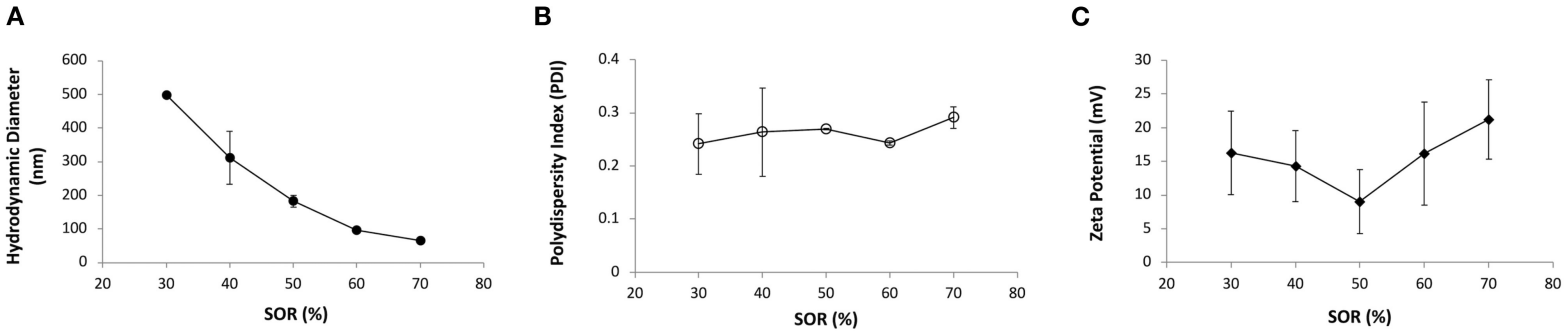
**(A)** Average hydrodynamic diameter (nm, full circles), **(B)** polydispersity index (PDI, empty circles) and **(C)** zeta potential (mV, full diamonds) of nanoemulsions containing PEG 660 12-hydroxystearate at different surfactant-to-oil ratios (SOR).

Previous studies demonstrated that by using a low-energy nano-emulsification process, it is possible to tailor the nanoemulsions particle size by varying the surfactant-to-oil weight ratio (SOR) (24, 25). Figure 1 shows the nanoemulsion droplets diameter, polydispersity index and surface charge in relation to different SOR values, when using an oil phase composed of vitamin E and sunflower oil, PEG 660 12-hydroxystearate as a non-ionic surfactant, and an aqueous phase containing chitosan 0.5% w/v dissolved in 0.5% v/v of acetic acid (also other surfactants were preliminary screened, *i.e*., Cremophor and α-tocopherol polyethylene glycol succinate, TPGS; see Supplementary Figure 3). Nanoemulsions with droplet size below 200 nm and positive surface charge could be obtained for SOR above 50% (NE50). The surface charge of these nanoemulsions, measured as zeta potential, ranged from 9 to 21 mV and this was attributed to the presence of chitosan, a polymer capable of conferring positive charge to the nanoemulsion surface in a slightly acidic environment (26, 27).

Further dimension analysis of a selected nanoemulsion (NE50) obtained as previously described was carried out using the NTA method. The results (reported in Supplementary Figure 4) showed that the average particle size was 120.2 ± 3.5 nm. The measure also highlighted that 10% of the nanoemulsion population had a hydrodynamic diameter around 37.0 ± 1.0 nm (D10); D50 was instead 67.8 ± 7.1 nm, while 90% of particles had dimensions below 172.6 ± 7.3 nm (D90). The interesting aspect of this method was that, unlike the DLS which provides an average size based on scattering intensity more affected by the larger nanodroplets, NTA is a measurement in which each nanoparticle is individually analyzed and measured, which provides results based on the number distribution of particles (28). Moreover, knowing the volume of the suspension, which in the case was 1 ml, the NTA software was able to calculate the concentration of the nanodroplets which was 3.98·10^8^ ± 1.53·10^7^ particles/ml.

### 3.2. Preparation of the vaccine powders loaded with nanoemulsion and antigen

To manufacture a dry powder vaccine for *M. hyopneumoniae*, the adjuvant nanoemulsion was layered onto solid carrier particles with an innovative method of controlled wetting/drying under conditions aiming at preserving the properties of both the nanoemulsion and the antigen. Three crystalline solid pharmaceutical excipients were selected as carriers: mannitol, lactose, and calcium carbonate.

**Figure 2:**
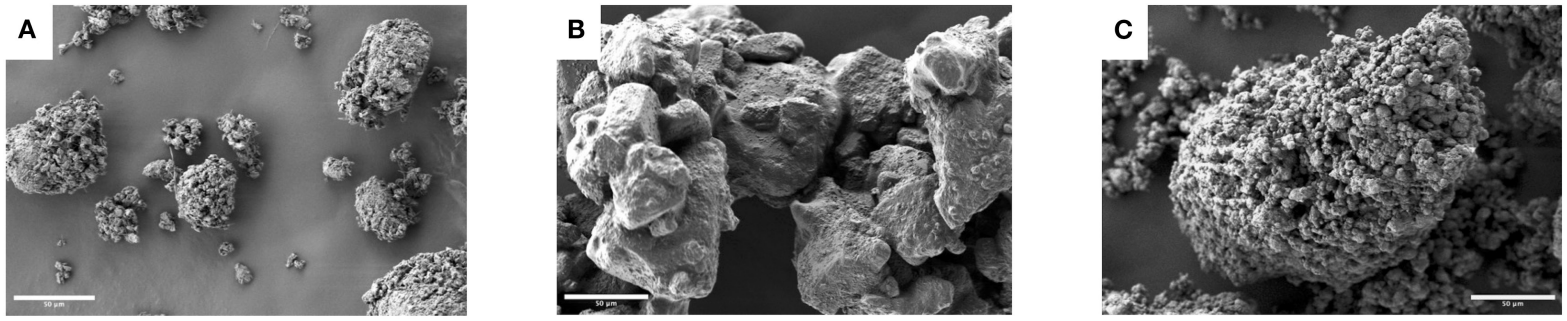
Scanning electron micrographs of solid carrier excipients coated with the nanoemulsion NE50: **(A)** mannitol, **(B)** lactose and **(C)** calcium carbonate (1,000× magnification, scale bars: 50 μm).

Scanning electron microscopy (SEM) images of the powders prepared by layering the NE alone onto these carriers are shown in Figure 2. SEM images show significant differences in terms of particle shape and morphology which are closely linked to the carrier powder characteristics and to the nanoemulsion drying process. From the pictures it can be observed that the NE layering causes the formation of agglomerates of the carrier powder, regardless of the excipient used.

Powders prepared using mannitol formed relatively small agglomerates with a spheroidal shape, showing cavities and a rough surface. Moreover, nanofibers with a thickness lower than 100 nm could be noticed, which physically bound the mannitol crystals (Figure 2A). These nanofibers were not present in the raw material or in the powders prepared with other excipients (see also Supplementary Figure 5 for a high magnification of the particle surface).

In the case of lactose (Figure 2B), the larger dimension of the starting typically tomahawk-shaped crystals was evident and consequently the formed agglomerates were larger than those observed with mannitol. The surface appeared more regular compared to the other materials, even if a roughness due to the coating of the particles appeared evident. The powders prepared with calcium carbonate showed the presence of agglomerates as well (Figure 2C). The agglomerate size was in between those of the other solid excipients tested, with an uneven surface attributed to closely packed and highly cohesive carrier particles. The crystallinity of the carriers was not affected by the layering of the nanoemulsion (data not shown).

**Figure 3:**
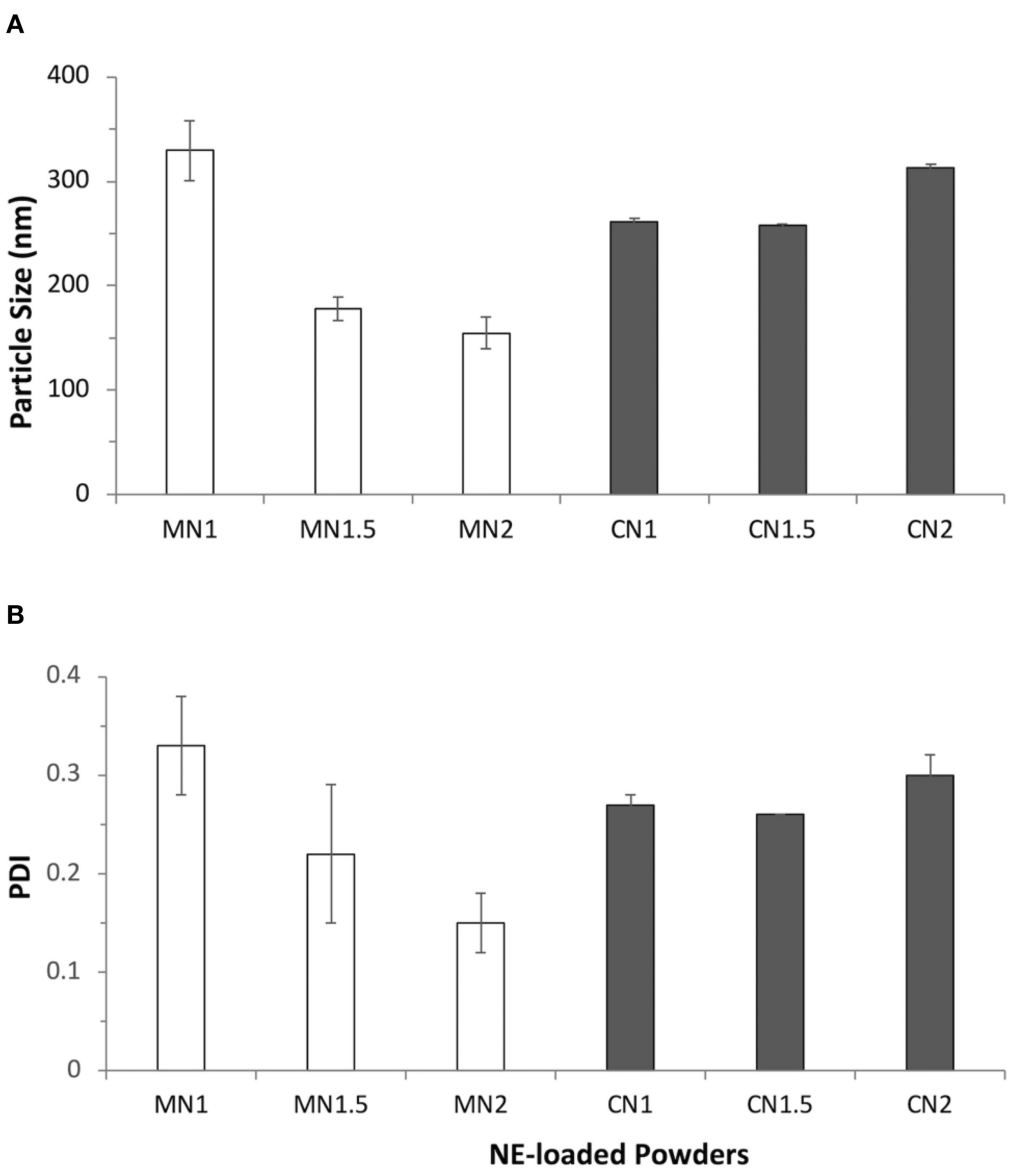
**(A)** Average size (nm) and **(B)** polydispersity index (PDI) of particles obtained after redispersion in water of dry powders obtained by layering nanoemulsions (NE50) on different solid carriers: mannitol (MN1, MN1.5, MN2; white bars) and calcium carbonate (CN1, CN1.5, CN2; gray bars) at different liquid-to-solid weight ratios.

The ability of the powders to reconstitute the nanoemulsion after dispersion in an aqueous medium was evaluated by dispersion in water and subsequent analysis of the colloidal dispersion was obtained. All lactose-based powders (LN1, LN1.5, LN2) showed poor dispersion abilities with average size above 500 nm and high polydispersity values (> 0.5) and hence were abandoned. The results obtained with the other excipients, presented in Figure 3, showed that the type of solid excipient and the ratio between solid carrier and nanoemulsion used for the preparation of the powder affected the nanoemulsion re-dispersion. For mannitol-based powders, a decrease in the redispersed nanoemulsion size (from 330 to around 160 nm) and polydispersity index (PDI) (from 0.330 to 0.150) was obtained by increasing the ratio between the nanoemulsion and the solid excipients. When calcium carbonate was used, the results were more consistent with a particle size in the range of 250–320 nm and a PDI lower than 0.3.

**Figure 4:**
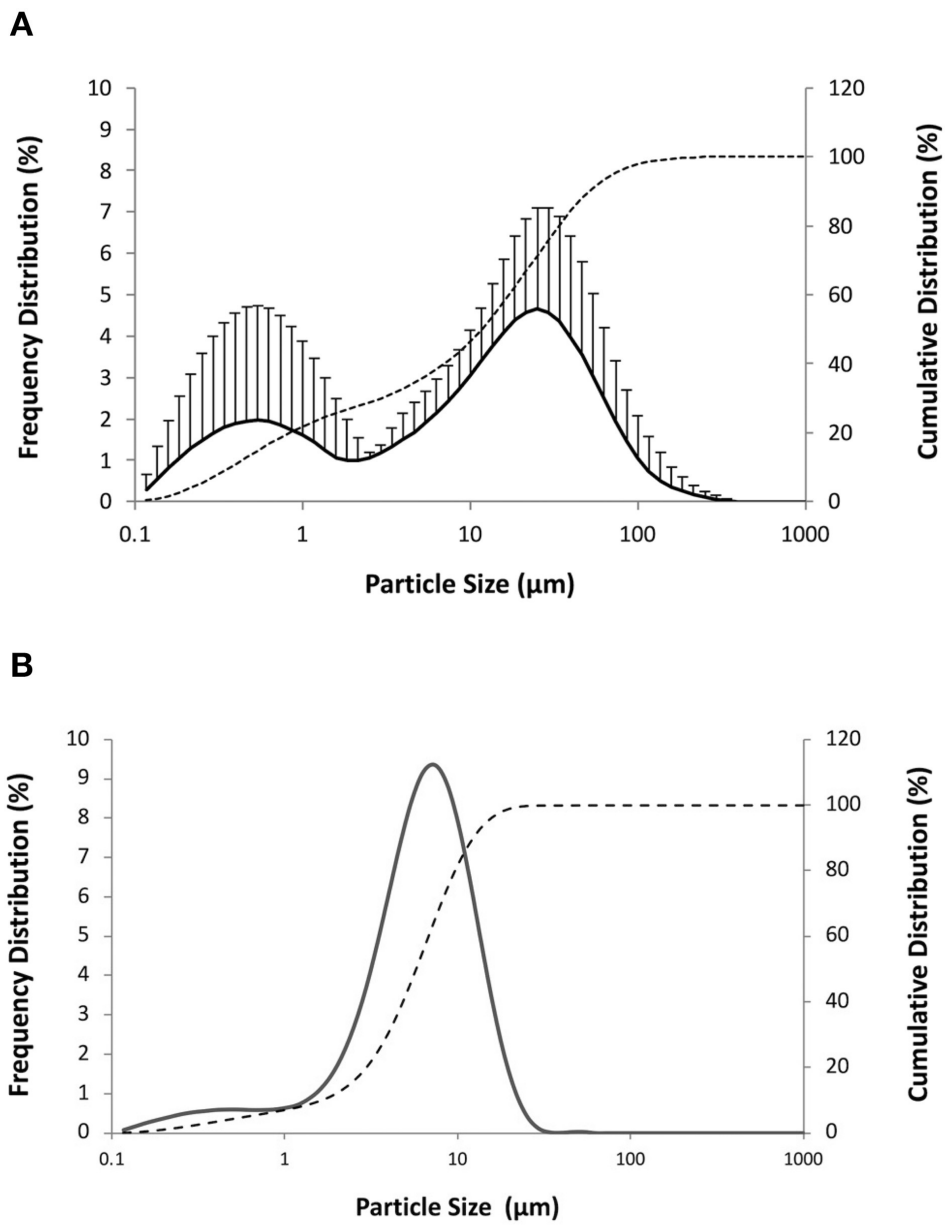
Cumulative (dashed line) and frequency particle size distribution (solid line) by volume of **(A)** redispersion in water of the dry powder vaccine NPV1 produced drying a mixture nanoemulsion NE50 and *M. hyopneumoniae* inactivate whole-cell concentrate (40:60) on mannitol (proportion liquid-to-solid carrier of 2:1 by weight) and **(B)** *M. hyopneumoniae* inactivated whole-cell concentrate.

Powders were then prepared using a mixture of whole-cell *M. hyopneumoniae* concentrate (1·10^10^ bacterins/ml in water) and the nanoemulsion NE50 in a volume ratio of 66:33 (NE/*M. hyopneumoniae*). Based on the indications from experiments with the nanoemulsion alone, the liquid dispersion of submicron particulate adjuvant and antigen was deposited onto the solid carrier using a liquid-to-solid ratio of 2:1 (NVP1, mannitol as solid carrier; NVP2, calcium carbonate as solid carrier). Once adsorbed onto the solid carrier, both the nanoemulsion and *M. hyopneumoniae* whole-cell concentrate should ideally be released in their original particle size distribution upon redispersion in biological fluids. The particle size distributions obtained by laser diffraction after the mannitol-based dry powder NPV1 redispersion are presented in Figure 4, which reports also for comparison the values obtained for the nanoemulsion NE50 and the inactivated whole-cell concentrate of *M. hyopneumoniae* measured alone. From Figure 4A, it can be appreciated that the redispersion in water of the dry powder vaccine presented in this example provided the redispersion of two populations of particles. One population with a peak close to 25 μm can be attributed to the *M. hyopneumoniae* antigen, as demonstrated by its particle size distribution presented in Figure 4B, where the peak of the distribution is slightly below 10 μm. The second population with a peak below 1 μm and centered around 550 nm was attributed to the submicronic particulate nanoemulsion adjuvant (Figure 4A). Similar results could be obtained with the dry powder vaccine powder obtained using calcium carbonate (data not shown). From these results, it appears that both the nanoemulsion and the antigen when redispersed present an increase in particle size, possibly as a result of partial aggregation of the two materials.

### 3.3. Performance of nasal vaccine powders with a nasal powder device

The NVP1 and NVP2 powders obtained layering respectively onto mannitol and calcium carbonate solid carriers both the nanoemulsion adjuvant and *M. hyopneumoniae* antigen were tested with a capsule-based nasal powder device suitable for the administration of the nasal powder vaccine to piglets (see Supplementary Figures 1, 2). The emitted dose was evaluated for the two powders using hypromellose capsules loaded with 25 mg of powder. The performance was also evaluated after storage of the capsules at room temperature and high temperature and relative humidity (45°C, 75% RH). Both formulations performed optimally with the selected nasal powder device, with TED above 90% (98.55 ± 1.54% TED for NVP1 and 94.77 ± 4.15% TED for NVP2). Storage at room temperature did not show any significant effect on TED. On the contrary, storage at high temperature and relative humidity for up to 14 days afforded a significant reduction of TED for the mannitol-based NVP1 formulation (82.96 ± 3.64% TED). Since the calcium carbonate-based nasal powder formulation appeared less sensitive to environmental conditions (99.14 ± 6.15% TED after 14 days), NVP2 was selected for the *in vivo* studies.

### 3.4. *In vivo* study in piglets

#### 3.4.1. Nasal powder vaccine effect in the nasal cavity

Piglets were assigned to three groups and vaccinated either intranasally with NVP2 administered with a powder device (IN group) or intramuscularly (IM group), while the control group (C group) received intranasally the powder formulation without the antigen.

**Figure 5:**
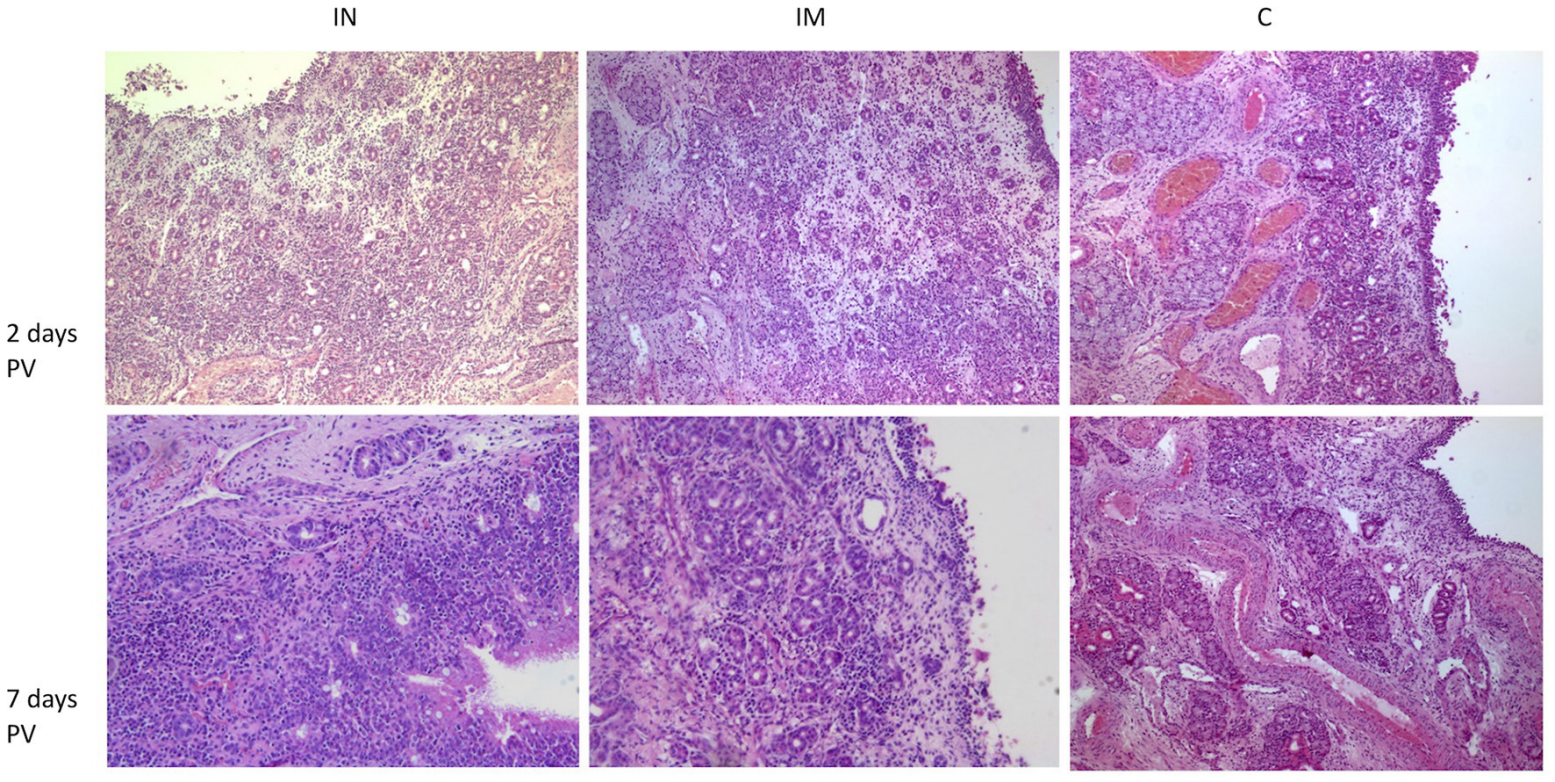
Histological features of nasal mucosa sections in the IN-vaccinated group, IM-vaccinated group and C group at 2 days and 7 days PV. H&E (100× magnification). IN group: at 2 days PV, epithelium, superficial and deep corium are diffusely infiltrated by lymphocytes, plasma cells and macrophages with superficial edema; at 7 days PV, within superficial corium, a diffuse and dense inflammatory infiltrate composed predominantly by numerous lymphocytes and plasma cells is present. Numerous intraepithelial lymphocytes are visible within epithelium. IM group: at 2 days PV, deep corium is multifocally infiltrated mainly by lymphocytes and plasma cells, while superficial corium is edematous; at 7 days PV, within superficial corium, a periglandular lymphoplasmacytic infiltrate is present; the epithelium is focally eroded. C group: at 2 days PV, within superficial corium, a multifocal lymphoplasmacytic infiltrate is present; at 7 days PV within superficial corium, a low number of lymphocytes and plasma cells surround glands and capillaries. IN, intranasally; IM, intramuscularly; C, control; PV, post-vaccination; H&E, hematoxylin and eosin.

Histology of the nasal tissue of the three groups at 2 and 7 days post-vaccination (days PV) is presented in Figure 5. The results of the histological scoring performed on the nasal mucosa biopsies are reported in the Supplementary Tables 2–4.

**Figure 6:**
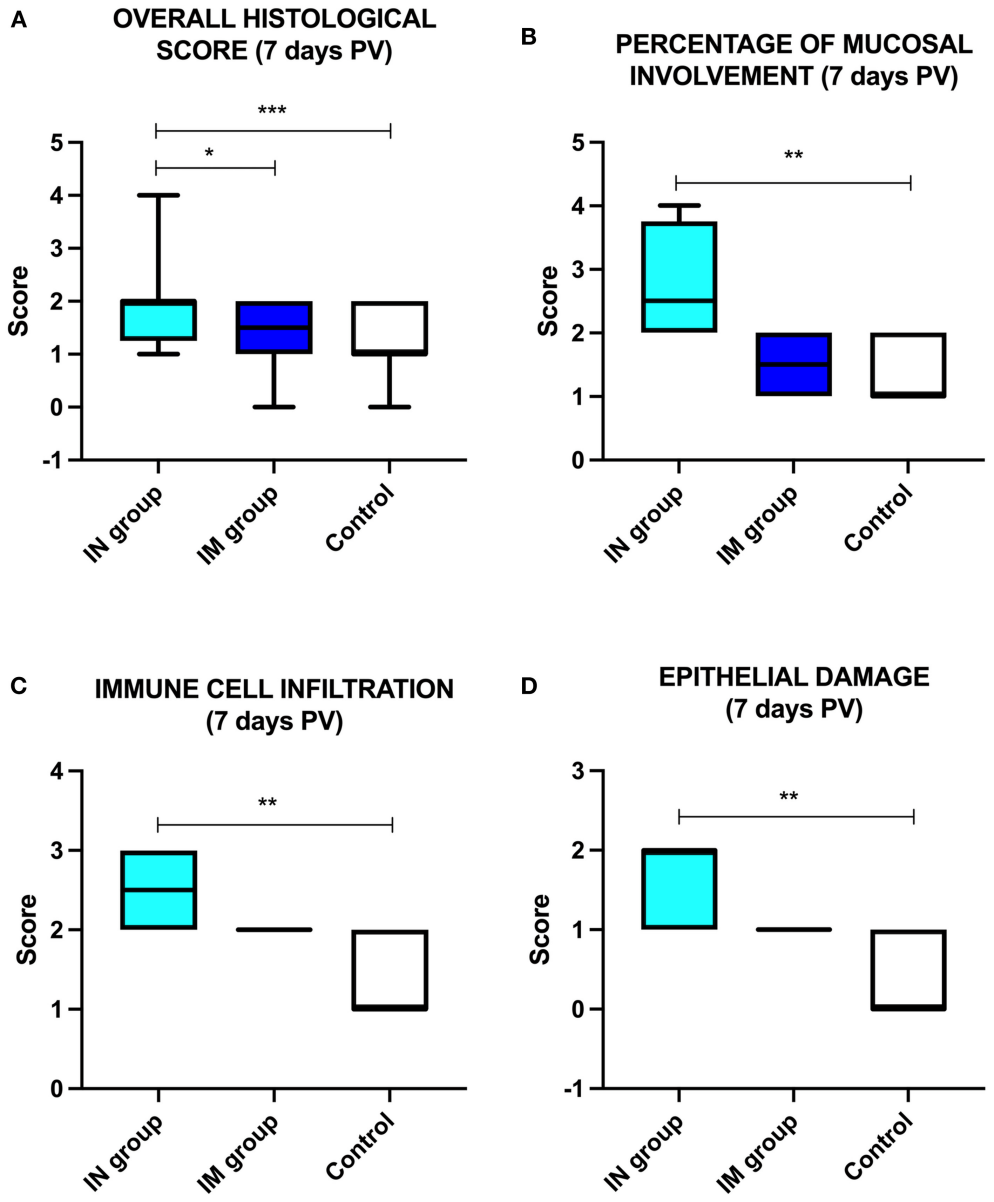
Histological analysis. Schematic representation of **(A)** the overall histological score in the control group, IN-vaccinated group (nano-adjuvanted dry powder vaccine) and IM-vaccinated group at 7 days PV; **(B)** percentage of mucosal involvement, **(C)** immune cell infiltration and **(D)** epithelial damage in the control group, IN-vaccinated group and IM-vaccinated group. *p < 0.05; **p < 0.01; ***p < 0.001. IN, intranasally; IM, intramuscularly; PV, post-vaccination.

The overall histological score showed statistically significant differences between the IN group and the control group and between the IN group and the IM group at 7 days PV (Figure 6A; ^***^p < 0.001 and ^*^p < 0.05, respectively) with higher values in the IN group. Regarding the single histological parameters considered, statistically significant differences were observed at 7 days PV between the IN group and the control group for the following parameters: percentage of mucosal involvement, infiltration of immune/inflammatory cells, and epithelial damage (Figures 6B–D; ^**^p < 0.01).

**Figure 7:**
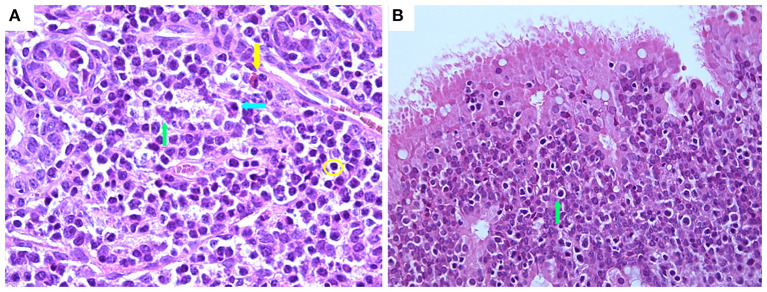
Histological features of nasal mucosa sections in the IN-vaccinated (nano-adjuvanted dry powder vaccine) group at 7 days PV. **(A)** Immune cell infiltrate within lamina propria mainly composed of lymphocytes (yellow circle), plasma cells (blue arrow) and macrophages (green arrow) with rare eosinophils (yellow arrow) (630× magnification); **(B)** numerous intraepithelial lymphocytes within epithelium (green arrow) (400× magnification). IN, intranasally.

Regarding the immune/inflammatory cell infiltrates within the mucosa, these were constituted in all groups mainly of mononuclear cells, specifically lymphocytes and plasma cells, and to a lesser extent by macrophages, which diffusely infiltrated the interstitium or formed perivascular aggregates within the corium or rarely extended into the deep mucosal layer (Figure 7A). Neutrophils were not observed in the different groups, while in the IN group at 7 days PV, numerous intraepithelial lymphocytes (IEL) were present (Figure 7B).

#### 3.4.2. Immune T and B cell responsiveness upon nasal vaccination

**Figure 8:**
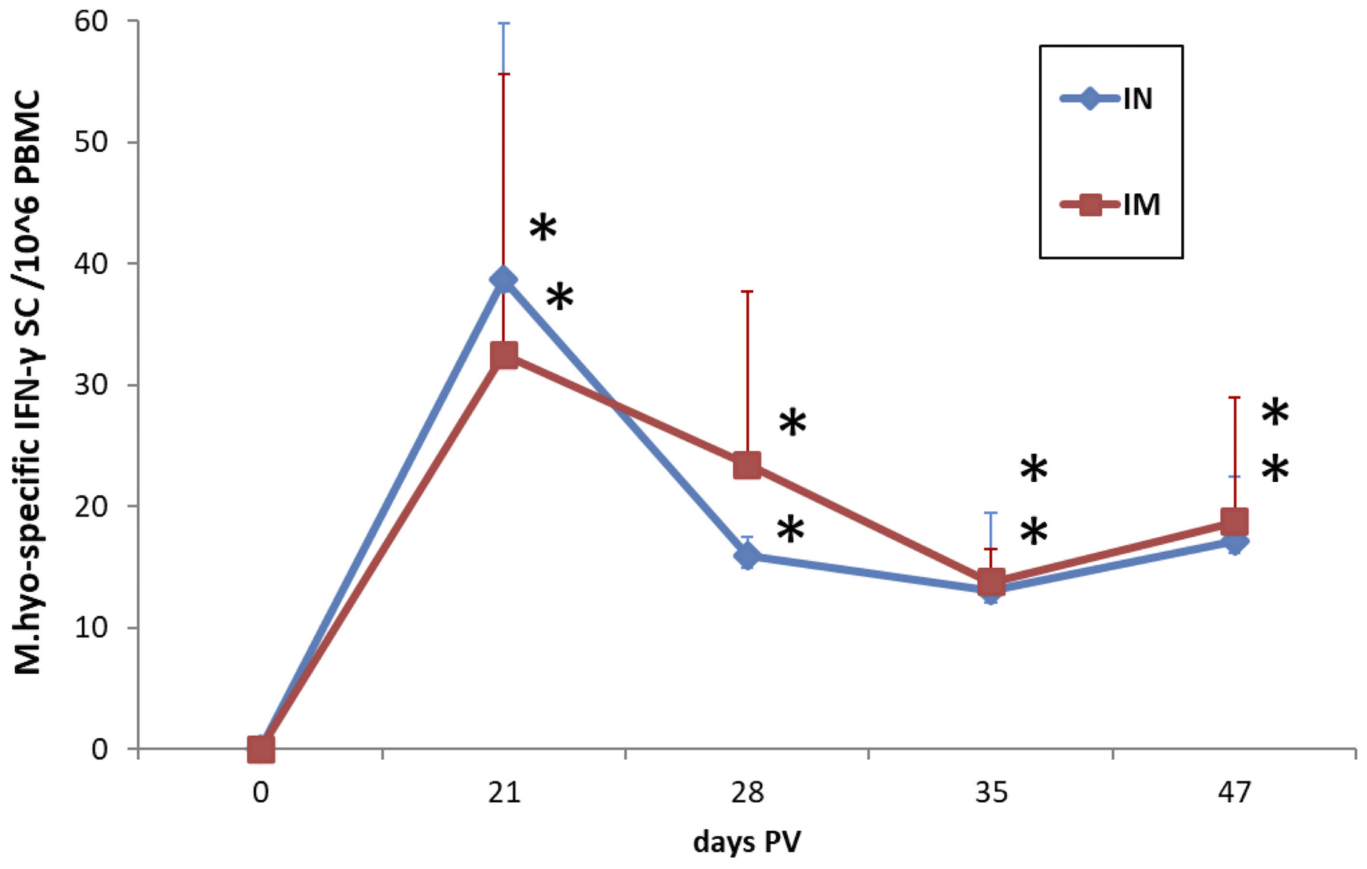
Frequencies of *M. hyopneumoniae*-specific IFN-γ secreting cells in PBMC of IN-vaccinated (nano-adjuvanted dry powder vaccine) and IM-vaccinated piglets. PBMC were recalled *ex vivo* with inactivated *M. hyopneumoniae* at a ratio of 100 bacterins/cell (~50 μg/ml) for 20 h. The levels are reported after subtraction of the values in the corresponding unstimulated cell samples and normalized to 10^6^ PBMC. Asterisks (*) indicate a statistically significant difference between the corresponding treatment group and the control group (*p* < 0.05). The mean value of the mock-vaccinated control group at 0 days PV (value = 0) was used as reference value for group comparisons throughout the study period. IN, intranasally; IM, intramuscularly; M.hyo, *M. hyopneumoniae*; SC, secreting cells; PV, post-vaccination.

IN vaccination with the dry powder vaccine induced a significant cellular response in terms of frequencies of *M. hyopneumoniae*-specific IFN-γ SC in PBMC at 21 days PV (*p* < 0.05), which was still detectable at 47 days PV (*p* < 0.05). The response was not significantly different from the response induced by IM vaccination with the same antigen (Figure 8).

**Figure 9:**
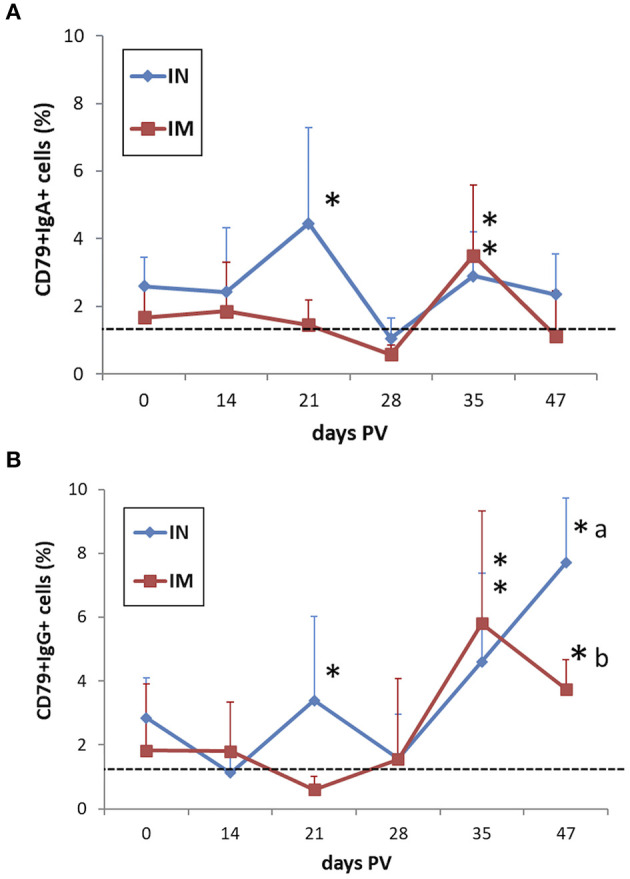
Frequencies of **(A)** CD79α+IgA+ and **(B)** CD79α+IgG+ B cells in *M. hyopneumoniae*-stimulated PBMC of IN-vaccinated (nano-adjuvanted dry powder vaccine) and IM-vaccinated piglets. PBMC were recalled *in vitro* with inactivated *M. hyopneumoniae* at a ratio of 100 bacterins/cell (~50 μg/ml) for 44 h. The levels are reported after subtraction of the corresponding values in unstimulated cell samples. Asterisks (*) indicate a statistically significant difference between the corresponding treatment group and the control group (*p* < 0.05). The mean value of the mock-vaccinated control group at 0 days PV (dashed line) was used as reference value for group comparisons throughout the study period. Different superscript letters indicate a statistically significant difference between the two treatment groups (*p* < 0.05). IN, intranasally; IM, intramuscularly; PV, post-vaccination.

The B cell response evaluated by quantifying the frequencies of CD79α+IgA+ cells and CD79α+IgG+ cells in PBMC after *in vitro* recall with *M. hyopneumoniae* showed increased levels of both cell fractions in IN-vaccinated pigs at 3 weeks PV in association with the peak of IFN-γ SC (*p* < 0.05). Both IN- and IM-vaccinated pigs showed a significant positive modulation of IgA and IgG-expressing cells at 35 days PV (*p* < 0.05) (Figures 9A, B). The CD79α+IgG+ cell reactivity was maintained in both vaccinated groups up to 47 days PV (*p* < 0.05) (Figure 9B).

## 4. Discussion

Commercial vaccines consisting of inactivated adjuvanted whole-cell preparations are widely applied worldwide in veterinary practice. In the countries with the largest pig population, vaccination for controlling *M. hyopneumoniae* infections is applied in more than 70% of the pig herds. The main advantages of vaccination include improvement of daily weight gain (2–8%) and feed conversion ratio (2–5%) and, sometimes, reduction in mortality rate. Additionally, shorter time to reach slaughter weight, reduced clinical signs, lung lesions and lower treatment costs are observed (29). Although protection against clinical pneumonia is often incomplete and vaccines do not prevent colonization (30), some studies indicate that the currently used vaccines may reduce the number of organisms in the respiratory tract (2) and may decrease the infection level in a herd (1). Using an experimental transmission model Meyns et al. (2) showed that, although vaccination against *M. hyopneumoniae* with a commercial vaccine significantly reduced the clinical symptoms and lung lesions in pigs, only a limited and non-significant reduction in the transmission of *M. hyopneumoniae* was achieved. As might be expected, they concluded that vaccination alone, and with the current vaccines will likely not be sufficient to eliminate *M. hyopneumoniae* from infected pig herds.

An “ideal” vaccine should induce effective immunity specific for the type of infection, have a long duration of immunity, protect also at the site of entrance of the pathogen, require minimal amount of adjuvant, demonstrate flawless safety and should be easy to administer (31). Most pathogens initially infect the host through the respiratory mucosal tissues (32). It is now well-established that intranasally administered vaccines can provide effective immunostimulation, both in terms of humoral and cell-mediated responses, especially if the vaccine is based on attenuated live cells or if the antigen is adjuvanted by means of an immunostimulant or a delivery system (33). Indeed, the NALT has a full range of immunocompetent cells, including B lymphocytes, CD4+ and CD8+ lymphocytes, phagocytic antigen-presenting cells and various subsets of dendritic cells (3).

In the present study, an innovative nasal vaccine powder is proposed by combining a nanoemulsion, a mucoadhesive polymer and the inactivated antigen. Indeed, sunflower oil and vitamin E are known to elicit immune responses (33, 34), while the cationic polysaccharide chitosan has series of appealing properties as vaccine excipient, providing mucoadhesion, enhancing mucosal permeability, controlling antigen release, and boosting adjuvant effects (10, 11). It was demonstrated that nanoparticles work as successful adjuvants since they act as delivery systems and/or immune modulators for vaccine applications, including mucosal vaccines (35). Recently, hybrid nanocapsules composed of an adjuvant lipid core and a cationic polysaccharide corona, *i.e*., squalene and polyglucosammine, and with size in the same range of the proposed nanoemulsions (200 nm) were successfully used to administer intramuscularly hepatitis B surface antigen and hemagglutinin of influenza virus, obtaining a significant enhancement of the immune response in mice (36). In a previous study, adjuvant oil-in-water nanoemulsions incorporating PEGylated surfactants and chitosan were shown to be biocompatible and able to diffuse through the mucus barrier. This latter feature was attributed to chitosan-mucin interactions that can create density inhomogeneity and an increase in the pore size within the mucous gel network, which enhances the mobility of PEGylated NEs (37). In the proposed vaccination strategy, O/W NEs with particle size below 200 nm, narrow particle size distribution and positive surface charge were manufactured. The nanoadjuvant was designed to exploit the above-mentioned features using immunostimulant excipients such as the sunflower oil and the cationic polysaccharide chitosan.

A further advantage of the formulation proposed in the present research was the use of a dry powder as a carrier for the combination of the nanoadjuvant and the antigen. Indeed, the use of dry powder formulations appears to be promising to provide physical, chemical, and microbiological stability to the vaccine product, potentially avoiding the need for preservatives, buffers and cold-chain storage transportation. At the same time, the use of dry powder formulations is challenging since nanometric-sized vectors and adjuvants, because of their size and composition, might be prone to aggregation (8) and commonly adopted drying processes are known to affect the size of sub-micron particles (38, 39).

Huang and co-workers proposed a dry powder nasal vaccine for influenza prepared by freeze-drying of whole inactivated influenza virus followed by milling and sieving and containing chitosan as mucoadhesive (40). Spray freeze-drying was proposed to produce anthrax nasal vaccine powder formulations containing a recombinant antigen of *Bacillus anthracis* combined with adjuvants such as a CpG-containing oligonucleotide (41) or a mast cell activator (42). Powder formulations offered an improved storage stability with respect to the liquid formulation and comparable toxin neutralizing antibodies to an intramuscular immunization with the same antigen. On the other hand, freeze-drying and milling have been successfully applied to the development of proprietary GelVac™ vaccine powders for nasal immunization against viral gastroenteritis (43). The formulation consists of norovirus-like particles and a mucoadhesive anionic polysaccharide derived from *Aloe vera* (GelSite^®^) which was shown to induce systemic and mucosal neutralizing antibodies in a dose-dependent manner after intranasal administration in guinea pigs, even in the absence of classic adjuvants (44). Notwithstanding, the freezing, spraying and drying processes, individually or combined, imply a series of physical stresses such as concentration of particulates and/or crystallization of solutes, dehydration and heating that could affect both antigen and sub-micron particulate vectors/adjuvant stability (45).

The disruptive technological solution presented here consists in the deposition of the liquid mixture of antigen and nanoadjuvant onto a solid carrier while blending the mixture, followed by drying under mild conditions, *i.e*., subjected to moderate heating and low vacuum (16). This process allows for mild conditions of drying preserving the integrity of both the nanoadjuvant and the antigen and allowing for their simultaneous release upon contact with the nasal mucus layer. The solid carriers selected were three common pharmaceutical excipients with different aqueous solubilities, namely mannitol, showing good aqueous solubility, calcium carbonate, insoluble in water, and lactose, with intermediate solubility but widely used as carrier for dry powder inhaler (DPI) products (46). Interestingly, only mannitol and calcium carbonate resulted in dry powder formulations able to redisperse the nanoadjuvant without aggregation, although dry powders based on mannitol showed a higher sensitivity to humidity during storage than those containing calcium carbonate as a carrier. This slightly affected the aerosolization performance of mannitol-based powders, as expected for more hydrophilic substances (47). Furthermore, nasal powder formulations containing calcium carbonate have been shown to improve nasal bioavailability of substances by increasing the residence time in the nasal cavity and are likely effective in improving delivery also in the case of vaccines (48).

The experimental dry powder nasal vaccine was tested *in vivo* in piglets, in a time interval when animals are susceptible to infections by *M. hyopneumoniae*. This time period which usually occurs from weaning onwards (during the weaning and growing phases), is extremely important for the development of an effective and lasting immunity in the animals (49). The significantly higher immune response detected in the nasal mucosa of IN-vaccinated piglets at 7 days PV compared to controls, characterized by local recruitment of lymphocytes, including intraepithelial lymphocytes, plasma cells and macrophages, testifies the efficacy of antigen recognition and stimulation of immune cells at the site of vaccine administration. The presence and recruitment of such immune cells confirm an efficient antigen presentation which in turn can trigger the activation of B and T lymphocytes able to mount an effector response and develop the immune memory against the pathogen.

The induction of both a local and systemic adaptive immune response in IN-vaccinated piglets was demonstrated by the increase of antigen-recalled IFN-γ SC in the blood after vaccination and the detection of significant levels up to 47 days PV. The response in IN-vaccinated pigs proved to be comparable with that observed in IM-vaccinated animals which represent a positive control as efficacious immune stimulation upon vaccination. The values observed in the IN-vaccinated group are also comparable with those observed in one-dose IM-vaccinated and needle-less intradermally (ID)-vaccinated pigs of a previous study (19). This further confirms the efficacy of the experimental dry powder IN vaccine in terms of induction of a systemic memory T cell immune response.

Regarding B cell responsiveness upon vaccination, it is known that IgA-expressing B lymphocytes play a fundamental role upon infection by respiratory pathogens because IgA represent the main immunoglobulin isotype acting at mucosal sites. In our study, *M. hyopneumoniae*-recalled B cells expressing secretory IgA showed increased levels at 21 days PV compared to the absence of modulation in IM-vaccinated animals, thus supporting the presence and activation of circulating mucosal B cells which can be subjected to specific tissue homing against the pathogen. In fact, the subsequent reduction in IN-vaccinated animals can be attributed to a distribution of such cells from blood to mucosal sites. The comparable recall at a later time-point (35 days PV) in both vaccinated groups suggests a later response in IM-vaccinated animals where the antigenic determinants may take longer to elicit a systemic detectable mucosal response.

A comparable course was observed for IgG+ B cells which proved to be more prone to be recalled in IN-vaccinated animals. This latter result demonstrates that activated antigen-specific B cells could be induced within 3 weeks from IN vaccination and be detected up to about 7 weeks PV. The further reduction of the mean values may be due to a distribution between the circulation and lymphatic organs while the gradual increase during the later phase of the study may be due to a prolonged activation of T helper cells after vaccination which can be involved in the secretion of IFN-γ and other Th1 cytokines which sustain IgG-expressing cells.

The slower induction of IgG-expressing B cells in IM-vaccinated pigs is in accordance with the slow onset of IgG in the blood upon *M. hyopneumoniae* antigen exposure (50–52). Indeed, it is known that the induction of circulating IgG is not a good indicator or predicting parameter of an effective immune response or clinical protection but rather an indicator of antigen encounter by immune cells. Therefore, IgA+ B cells can be more involved in the immune modulation and activation against *M. hyopneumoniae* upon IN vaccination with the experimental nano-adjuvanted dry powder vaccine developed in the present study. These cells have the potential to mature into plasma cells homing to mucosal tissues such as the lungs and the upper respiratory tract, and thus constitute, together with IgG+ B cells, the memory B cell (MBC) pool during the lifespan of the animal to counteract *M. hyopneumoniae* infections (53–55).

## 5. Conclusions

A dry powder nasal vaccine combining a nanoemulsion-based adjuvant and *M. hyopneumoniae* bacterins was produced by using an innovative production method, based on the controlled drying of the liquid mixture on solid particles of conventional pharmaceutical excipients such as mannitol and calcium carbonate. Once dispersed in an aqueous environment, the dry powder vaccine was demonstrated to be able to reconstitute the initial components enabling the simultaneous delivery of antigen and adjuvant. The vaccine was tested in piglets by means of a nasal insufflator demonstrating both the local recruitment of immune cells and the induction of *M. hyopneumoniae-*specific immune cells to an extent comparable with the intramuscular immunization with the same antigen.

Therefore, the nasal vaccine developed may be considered as a prototype, with the potential, when combined with a multi-dose delivery device suitable for field immunization campaigns, yet to be developed, to be a viable alternative to traditional vaccination strategies for veterinary immunization, with the advantage of the improved chemical and microbiological stability provided by a dry powder formulation.

## Data availability statement

The raw data supporting the conclusions of this article will be made available by the authors, without undue reservation.

## Ethics statement

The animal study was reviewed and approved by the Ethical Committee Organismo Preposto al Benessere Animale (OPBA) of the Department of Veterinary Science, University of Parma, Parma, Italy.

## Author contributions

RB, PM, PB, EC, and FS contributed to conception and design of the study. FC, NA, and AB contributed to the acquisition, analysis, and interpretation of data related to the formulation. EC, LF, and GEM contributed to the acquisition, analysis, or interpretation of data related to the experimental *in vivo* study. FS wrote the first draft of the manuscript. EC, LF, GEM, and FB wrote sections of the manuscript. All authors contributed to manuscript revision, read, and approved the submitted version.
