# Supplementary material for: Nano-adjuvanted dry powder vaccine for the mucosal immunization against airways pathogens
Source: Front Vet Sci. 2023 Mar 14;10:1116722. doi: 10.3389/fvets.2023.1116722 (PMC10043307; doi:10.3389/fvets.2023.1116722)
Supplement: Supplementary file 1 [file Data_Sheet_1.DOCX]

Supplementary Material

# Nasal Powder Device

Vaccine powders were loaded into a nasal powder device (Monodose Nasal Insufflator, MIAT, Milano, Italy, Supplementary Figure 1) for administration to piglets during the *in vivo* study (Supplementary Figure 2).


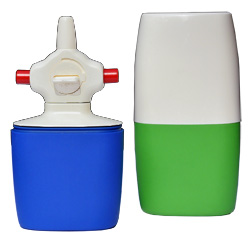


**Supplementary Figure 1.** MIAT Monodose Nasal Insufflator.

The device nosepiece was suitable for the administration in piglets as shown in the picture reported here.


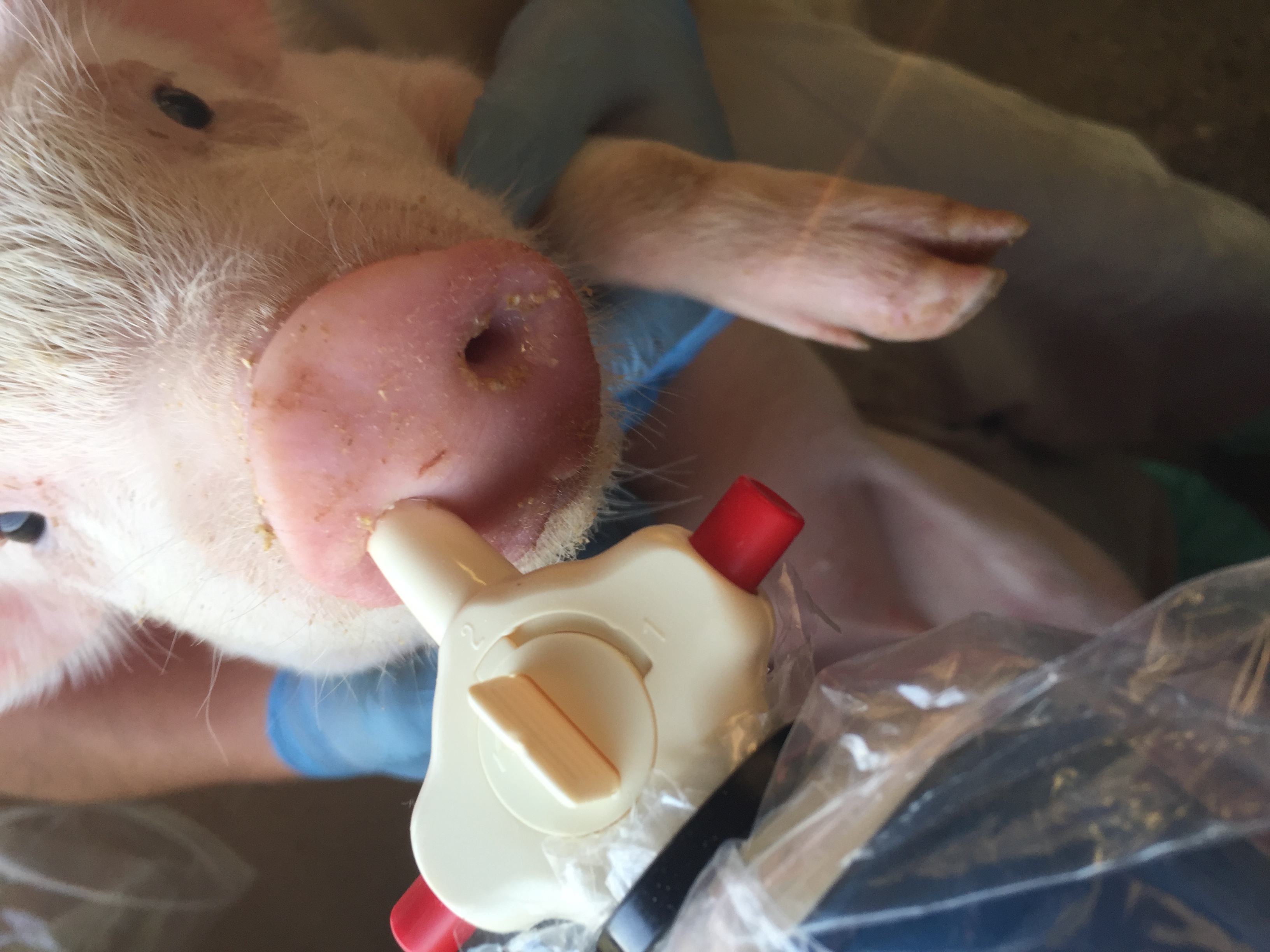


**Supplementary Figure 2.** Powder nasal insufflator use with piglets for nasal powder vaccine administration.

# Surfactant Screening for the Low-Energy Nanoemulsification Process

Supplementary Figure 3 shows the nanoemulsion droplets diameter and polydispersity index (PDI) in relation to different SOR values, when using an oil phase composed of vitamin E and sunflower oil, Cremophor (Supplementary Figure 3A) or TPGS (Supplementary Figure 3B) as a nonionic surfactant, and an aqueous phase containing chitosan 0.5% w/v dissolved in 0.5% v/v of acetic acid.

**Supplementary Figure 3.** Dependence of average size (solid line) and PDI (dashed line) on the surfactant-to-oil ratio (SOR) of nanoemulsions prepared using (A) Cremophor or (B) TPGS as non-ionic surfactants.

While in the case of Cremophor there is a significant reduction in average size and variability when SOR increases above 50%, the reduction in average particle size is more progressive for TPGS-containing nanoemulsions. For both non-ionic tensioactives, size reduction could be attained only at high SOR ratios and zeta potential (not shown) showing highly variable values, ranging from -2.61 to 16.32 mV.

Nanoparticle Tracking Analysis (NTA)

Particle size analyses for NE50 nanoemulsion in terms of intensity distribution (Supplementary Figure 4A) and particles concentration (Supplementary Figure 4B) versus particle size. The results showed that the average particle size was 120.2 ± 3.5 nm.

**A B**


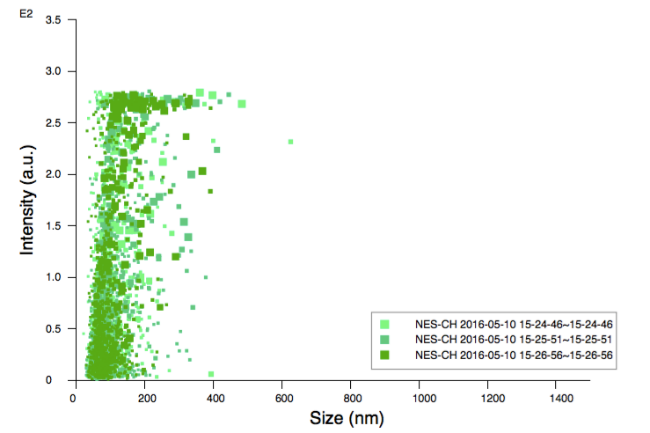

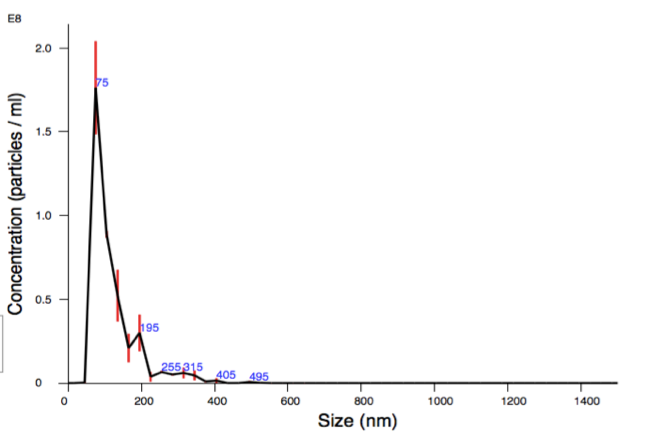


**Supplementary Figure 4.** Nanoparticle tracking analysis of NE50 in terms of Intensity vs. Size (A) and Concentration vs. Size (B). Error bars indicate standard error of the mean.

# SEM images of the surface of powders loaded with nanoemulsion

High magnification SEM images of the surface of powders obtained by layering nanoemulsion NE50 onto solid carrier particles with an innovative method of controlled wetting/drying (Supplementary Figure 5).

**Supplementary Figure 5.** Scanning electron micrographs of solid carrier excipients coated with nanoemulsion: mannitol (A), lactose (B) and calcium carbonate (C). Magnification factor 5,000×.

# Histological analysis performed on nasal mucosa biopsies

The histological scoring parameters are reported in Supplementary Table 1.

**Supplementary Table 1.** Histological scoring parameters for the evaluation of the nasal mucosa biopsies.

| **Histological feature** | **Score** | **Description** |
| --- | --- | --- |
| Percentage of mucosal involvement | 0 | 0% |
|  | 1 | 1–25% |
|  | 2 | 26–50% |
|  | 3 | 51–75% |
|  | 4 | 76–100% |
| Localization of immune/inflammatory cells | 0 | None |
|  | 1 | Epithelium |
|  | 2 | Epithelium and superficial corium |
|  | 3 | Transmural |
| Grade of immune/inflammatory response | 0 | None |
|  | 1 | Mild |
|  | 2 | Moderate |
|  | 3 | Severe |
| Glandular damage | 0 | None |
|  | 1 | 1/3 Glands damaged |
|  | 2 | 2/3 Glands damaged |
|  | 3 | Glands absent, epithelium present |
| Epithelial damage | 0 | None |
|  | 1 | Focal erosion |
|  | 2 | Locally extensive erosion |
|  | 3 | Ulceration |

The histological scoring of nasal mucosal biopsies for IN, IM and C groups are reported in Supplementary Tables 2, 3 and 4, respectively.

**Supplementary Table 2.** Histological scores of the intranasally-vaccinated group (IN group).

| ID  Number | Days PV | Percentage of mucosal involvement | Localization of immune/inflammatory cells | Grade of immune/inflammatory response | Glandular damage | Epithelial damage | Immune/inflammatory cells* |
| --- | --- | --- | --- | --- | --- | --- | --- |
| **5** | **2** | 2-0-2-2 | 2-0-2-2 | 2-0-2-2 | 1-0-1-1 | 0-0-0-0 | Lymphocytes ++ Plasma cells ++  Macrophages ++  Eosinophils + |
| **9** | **2** | 3-3-3-3 | 2-2-3-2 | 2-2-2-3 | 1-1-1-1 | 1-2-2-2 | Lymphocytes +++ Plasma cells +++  Macrophages ++ Eosinophils + |
| **16** | **7** | 4-3-3-4 | 2-2-3-2 | 3-3-3-3 | 2-1-1-1 | 1-2-2-1 | Lymphocytes +++ Plasma cells +++  Macrophages ++ Eosinophils + |
| **17** | **7** | 2-2-2-2 | 2-2-2-2 | 2-2-2-2 | 1-1-1-1 | 1-2-2-2 | Plasma cells +++  Lymphocytes +++ Macrophages ++  Eosinophils + |
| **18** | **47** | 2-1-1-1 | 2-2-2-1 | 2-1-1-2 | 0-0-0-0 | 0-0-0-0 | Plasma cells +++  Lymphocytes ++  Macrophages ++ Eosinophils + |
| **2** | **47** | 3-4-3-3 | 2-2-2-2 | 2-3-2-2 | 1-2-1-1 | 0-0-0-0 | Lymphocytes ++ Plasma cells ++  Macrophages ++  Eosinophils + |
| **11** | **47** | 2-2-2-2 | 2-2-2-1 | 1-1-1-1 | 0-0-0-0 | 0-0-0-0 | Plasma cells +++  Lymphocytes ++  Macrophages ++  Eosinophils + |
| **12** | **47** | 1-1-1-1 | 2-2-2-1 | 1-1-1-1 | 0-0-0-0 | 0-0-0-0 | Eosinophils ++++  Lymphocytes +  Macrophages + |

PV: post-vaccination. * Only detected cells are reported.

**Supplementary Table 3.** Histological scores of the intramuscularly-vaccinated group (IM group).

| ID  Number | Days PV | Percentage of mucosal involvement | Localization of immune/inflammatory cells | Grade of immune/inflammatory response | Glandular damage | Epithelial damage | Immune/inflammatory cells* |
| --- | --- | --- | --- | --- | --- | --- | --- |
| **8** | **2** | 2-2-2-2 | 2-2-2-2 | 1-1-1-1 | 0-0-1-0 | 0-0-0-0 | Lymphocytes +++  Plasma cells ++ Macrophages ++  Eosinophils + |
| **14** | **7** | 2-1-2-1 | 2-2-2-2 | 2-2-2-2 | 1-1-0-0 | 1-1-1-1 | Lymphocytes ++  Plasma cells ++  Macrophages ++  Eosinophils + |
| **6** | **47** | 2-2-2-2 | 2-2-2-2 | 2-1-2-1 | 1-0-0-1 | 0-0-0-0 | Plasma cells ++++  Macrophages ++  Lymphocytes ++  Eosinophils + |
| **10** | **47** | 2-2-1-1 | 2-2-2-2 | 1-1-1-1 | 0-0-0-0 | 0-0-0-0 | Lymphocytes ++  Plasma cells ++  Macrophages ++  Eosinophils + |
| **13** | **47** | 1-1-1-1 | 2-2-2-2 | 1-1-1-1 | 0-0-0-0 | 0-0-0-0 | Lymphocytes +++  Plasma cells +++  Macrophages ++  Eosinophils + |
| **15** | **47** | 2-1-4-2 | 2-1-2-2 | 2-1-3-2 | 0-0-1-2 | 0-0-0-0 | Lymphocytes +++  Plasma cells +++  Macrophages ++  Eosinophils + |

PV: post-vaccination. * Only detected cells are reported.

**Supplementary Table 4.** Histological scores of the control group (C group).

| ID  Number | Days PV | Percentage of mucosal involvement | Localization of immune/inflammatory cells | Grade of immune/inflammatory response | Glandular damage | Epithelial damage | Immune/inflammatory cells* |
| --- | --- | --- | --- | --- | --- | --- | --- |
| **1** | **2** | 2-3-2-1 | 2-2-2-1 | 1-2-1-1 | 0-0-1-0 | 0-1-0-0 | Lymphocytes +++  Plasma cells ++  Macrophages +  Eosinophils + |
| **4** | **2** | 3-1-2-3 | 2-2-2-2 | 2-1-1-2 | 0-0-1-1 | 1-0-0-1 | Lymphocytes ++  Plasma cells ++  Macrophages ++  Eosinophils + |
| **3** | **7** | 2-1-2-2 | 2-2-2-2 | 2-1-2-1 | 1-1-1-0 | 0-0-0-0 | Lymphocytes +++  Plasma cells +++  Macrophages ++  Eosinophils + |
| **7** | **7** | 1-1-1-1 | 2-1-1-1 | 2-1-1-1 | 1-1-0-0 | 1-1-0-1 | Lymphocytes +++ Plasma cells +++  Macrophages ++  Eosinophils + |

PV: post-vaccination. * Only detected cells are reported.

.
